# Novel and unique domains in aminoacyl-tRNA synthetases from human fungal pathogens *Aspergillus niger*, *Candida albicans* and *Cryptococcus neoformans*

**DOI:** 10.1186/1471-2164-15-1069

**Published:** 2014-12-05

**Authors:** Manish Datt, Amit Sharma

**Affiliations:** Structural and Computational Biology group, International Center for Genetic Engineering and Biotechnology (ICGEB), New Delhi, 110067 India

**Keywords:** Aminoacyl-tRNA synthetases, Domain organization, Drug targets, Fungal pathogens

## Abstract

**Background:**

Some species of fungi can cause serious human diseases, particularly to immuno-compromised individuals. Opportunistic fungal infections are a leading cause of mortality, and present an emerging challenge that requires development of new and effective therapeutics. Aminoacyl-tRNA synthetases (aaRSs) are indispensable components of cellular protein translation machinery and can be targeted for discovery of novel anti-fungal agents.

**Results:**

Validation of aaRSs as potential drug targets in pathogenic microbes prompted us to investigate the genomic distribution of aaRSs within three fungi that infect humans – *A. niger*, *C. albicans* and *C. neoformans*. Hidden Markov Models were built for aaRSs and related proteins to search for homologues in these fungal genomes. Here, we provide a detailed and comprehensive annotation for 3 fungal genome aaRSs and their associated proteins. We delineate predicted localizations, subdomain architectures and prevalence of unusual motifs within these aaRSs. Several fungal aaRSs have unique domain appendages of unknown function e.g. *A. niger* AsxRS and *C. neoformans* TyrRS have additional domains that are absent from human homologs.

**Conclusions:**

Detailed comparisons of fungal aaRSs with human homologs suggest key differences that could be exploited for specific drug targeting. Our cataloging and structural analyses provide a comprehensive foundation for experimentally dissecting fungal aaRSs that may enable development of new anti-fungal agents.

**Electronic supplementary material:**

The online version of this article (doi:10.1186/1471-2164-15-1069) contains supplementary material, which is available to authorized users.

## Background

Fungal infections are emerging as a leading cause of human mortality in hospital settings [1–5]. Recent trends suggest that select species of fungi have the ability to take advantage of debilitated immune system to cause mortality [1–5]. Data suggest that diseases caused by opportunistic fungi result in scores of human deaths worldwide, and hence containment of human fungal infections is a pressing challenge [1–5]. The pathogenesis of such opportunistic fungi is a complex phenomenon since these organisms more often than not lack dedicated ‘virulence’ factors [1–5]. Therefore, understanding cellular and molecular attributes of these fungal pathogens is indispensible towards developing effective therapeutics.

*Aspergillus*, *Candida*, and *Crytococcus* are the three most common genus of fungi associated with infectious diseases in humans [1–5]. Candidiasis or thrush is the most prevalent fungal infection in humans, commonly caused by *C. albicans. Candida* is generally present on the skin and mucous membrane and does not cause infection, however invasive candidiasis occurs when *Candida* enters the bloodstream and spreads throughout the body [6]. Immuno-compromised (as in case of cancer and AIDS patients) individuals are particularly prone to *C. albicans* infections [7]. *Aspergillus* species causes second most common fungal infections in humans [8]. *A. fumigates* is the most virulent species in this genus, and cases of pulmonary aspergillosis have been reported resulting from *A. niger* infections as well [9]. Cryptococcosis is a fungal disease caused primarily by the two species *Cryptococcus neoformans* and *Cryptococcus gattii* [10]. *C. neoformans* is present in soil and spreads through microscopic airborne fungal spores [11]. *C. neoformans* infection increases the probability of re-hospitalization of AIDS patients who otherwise show positive response to anti-retroviral therapy [7, 12]. *A. niger* and *C. albicans* belong to Ascomycota phylum while *C. neoformans* belongs to Basidomycota phylum within the fungal kingdom. Genome sizes for *A. niger*, *C. albicans*, and *C. neoformans* are ~33 Mb (19 chromosomes), ~14 Mb (8 chromosomes), and ~19 Mb (14 chromosomes). Predicted number of ORFs (open reading frames) in *A. niger*, *C. albicans*, and *C. neoformans* are ~14000, ~6200, and ~6500. In addition to genome sequencing, transcriptomics analyses for these three fungi are also available [13–15].

Aminoacyl-tRNA synthetases (aaRSs) drive protein translational machinery by catalyzing the addition of amino acids to cognate tRNA [16, 17]. Structural studies have confirmed their modular architecture, with separate domains for aminoacylation and tRNA binding [16–18]. Based on their structural fold and mode of tRNA binding these enzymes have been classified into class I and II aaRSs [16–18]. In addition to performing translational activities, these proteins also localize within mitochondria and apicoplasts to participate in synthesis of proteins encoded by genomes within organelles [16–21]. Aside from their translational functions, aaRSs are implicated in various non-canonical functions such as gene transcription, mRNA translation, inflammation, and immune response [16–21]. Therefore, aaRSs constitute a very important subset of proteins in any genome, and inhibition of their enzymatic activity can be deleterious for the host. Ever since the potential of malaria parasitic aaRSs for exploration as new drug targets has been highlighted [19, 22], intense efforts from many groups have yielded an array of druggable malaria parasite aaRSs [23–35]. More recently, protein translation components from other eukaryotic pathogens like *Leishmania* [36] and *Toxoplasma* [21] have also been explored. Hence, experimental dissection of critical translation components like aaRSs is on high priority as one avenue of novel target discovery in pathogen biology. Dearth of structural characterization of larger number of fungal aaRSs severely restricts scope for targeted development of new anti-aaRS drugs, despite the acceptance of aaRSs as druggable targets. In context of this work, *C. albicans* SerRS is the only full-length aaRS from these three fungal genomes for which crystal structure has been solved [37]. Here, we have studied aaRSs from *A. niger*, *C. albicans* and *C. neoformans* using profile-based Hidden Markov Models (HMM). In addition, homologs for aaRS-related proteins such as the editing domains (AlaX) and D-tyrosyl-deacylase (DTD), Gln and Asn amidotransferases, and aaRS-associated P43 protein have also been identified. Protein sequences of thus identified aaRSs have been comprehensively annotated in context of their domain organization. We discovered novel domain appendages in several fungal aaRSs that are absent from human homologs. Our results highlight key structural attributes of fungal aaRSs that could be exploited for drug targeting to combat mycoses in humans.

## Methods

### Retrieval of fungal genome sequences and aaRS dataset

Computationally translated open reading frames (ORFs) for *A. niger* and *C. albicans* were downloaded from their corresponding genome databases available at http://www.aspergillusgenome.org/ and http://www.candidagenome.org/ respectively. Similarly, ORF sequences for *C. neoformans* were retrieved from FungiDB [38]. To annotate aaRSs and aaRS-associated proteins, a dataset of annotated aaRSs and associated proteins was prepared from the UniProt database [39]. Our dataset included protein sequences of human aaRSs, human aaRS trans-editing domain (DTD, AlaX), Gln and Asn amidotransferases, aaRS associated protein P43 and Ybak protein from *E. coli*. The UniProt accession numbers for sequences in the dataset are given in supplementary Additional file 1: Table S1.

### Generation of Hidden Markov Models (HMMs), protein annotation and modeling

All protein sequences from our dataset were individually used to perform BLAST searches against non-redundant databases available at NCBI. Top 1000 homologs from each BLAST search were saved for further analyses as we reasoned that these may effectively capture the evolutionary diversity at different positions within aaRSs, thus facilitating generation of robust HMMs. For each aaRS, HMMs were generated using the homologs identified from BLAST search. Sequence Alignment and Modeling Software System (SAM) [40] was used to generate MSA, and HMMER package was used for building profile HMM for each MSA. Significantly similar matches for each HMM profile were identified within the genomic sequences from the three fungi. Pfam [41] domains were assigned to predicted aaRS sequences from fungal genomes. Sub-cellular localization for sequences showing significant HMM search score was annotated using Wolf PSORTb webserver using default parameters optimized for fungal proteins [42]. For a query protein sequence, this server gives a relative score to each sub-cellular location and the highest scoring compartment has been used here to annotate aaRSs. MitoProt [43] and TagetP [44] servers were used to identify signal sequences within the predicted mitochondrial aaRSs. Sequence alignment of aaRSs was performed using T-coffee program with default parameters [45]. Structural conservation was mapped onto the sequence alignment using Expresso options available at http://tcoffee.crg.cat/. Homology modeling for protein sequences was performed using Phyre2 server [46] and PyMol [47] was used for structure visualization and analyses.

## Results and discussion

**Figure 1:**
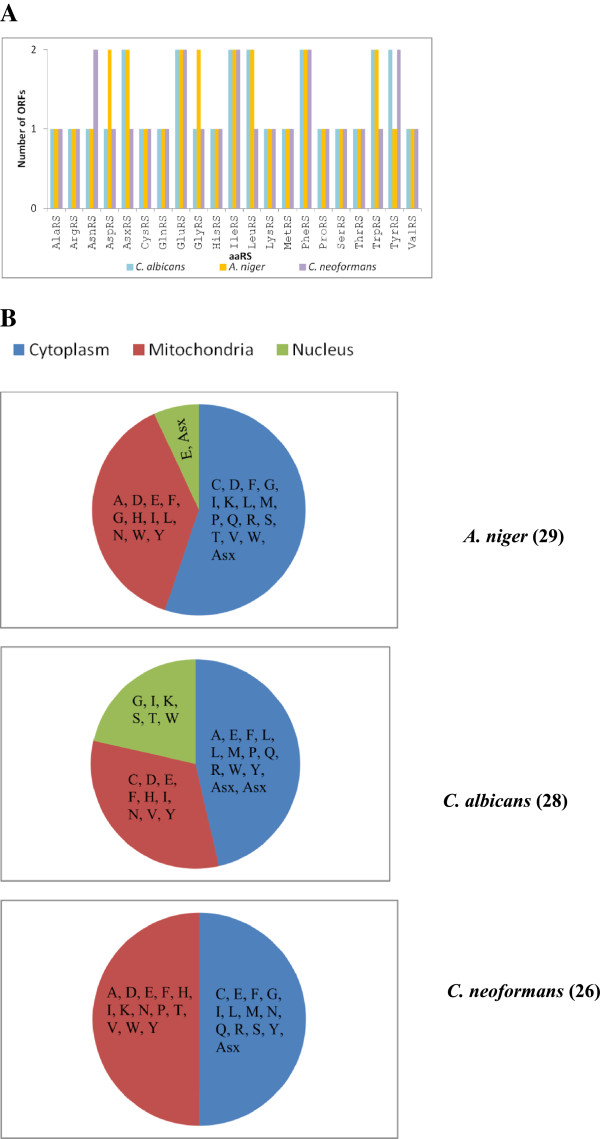
**Hidden Markov Model (HMM) based prediction of aaRSs in fungal genomes. (A)** Number of predicted aminoacyl-tRNA synthetases (aaRSs) in the three fungal genomes. **(B)** Predicted distribution of aaRSs in the sub-cellular compartments of the three fungal pathogens. The aaRSs are denoted by the single letter code for their corresponding amino acid and Asx refers to non-discriminating aspartyl-tRNA synthetase.

We have employed a profile HMM-based search to computationally identify aaRSs in the three fungal genomes. Our results show that there are a total of 29 aaRSs in *A. niger*, 28 in *C. albicans* and 26 in *C. neoformans* (Tables 1, 2 and 3). This exercise enabled us to annotate aaRSs specific for all 20 amino acids in these fungi (Tables 1, 2 and 3). All predicted aaRSs had highly significant e-values based on profile HMM searches. Functional annotation of these aaRSs was cross-validated using BLAST searches against conserved domain database (CDD) at NCBI. Amongst the three fungi, maximum variation in the full length of aaRSs was observed in *A. niger* genome with the largest and smallest proteins being IleRS (212883-mRNA) with 1524 residues and TrpRS (209919-mRNA) with 391 residues respectively (Table 1). Interestingly, for some aaRSs there exist multiple enzymes for charging tRNAs that are specific to a particular amino acid (Figure 1). Our results show that there are 8 *A. niger* aaRSs that are present in two versions (AspRS, GluRS, GlyRS, IleRS, LeuRS, PheRS, TrpRS and AsxRS), 7 *C. albicans* aaRSs that are present in two versions (GluRS, IleRS, LeuRS, PheRS, TrpRS, TyrRS and AsxRS), and 5 *C. neoformans* aaRSs that are present in two versions (AsnRS, GluRS, IleRS, PheRS and TyrRS, Figure 1A). These two versions of aaRSs would presumably facilitate localization in different sub-cellular compartments e.g. in cytoplasm and mitochondria. Comparisons amongst the three fungi show GluRS, IleRS, LeuRS and PheRS to be the common set of two-copy enzymes. To analyze compartmentalization of aaRSs, sub-cellular localization was calculated for all predicted aaRSs. Expectedly, this showed that whenever two same amino acid aaRSs were present, one was predicted to be cytoplasmic while the other mitochondrial (Tables 1, 2 and 3). We then were able to compile predicted sub-cellular distribution for all aaRSs (Figure 1B), along with putative signal sequences for mitochondrial aaRSs (Additional file 1: Figure S1). Out of total 29 *A. niger* aaRSs – 16, 11, and 2 aaRS(s) were predicted to (co-) localize in cytoplasm, mitochondria and nucleus (Figure 1B and Table 1). In *C. albicans* 13, 9 and 6 aaRSs were predicted to be cytoplasmic, mitochondrial and nuclear (Figure 1B and Table 2). Finally, *C. neoformans* aaRSs were predicted to be equally partitioned between cytoplasm and mitochondria with 13 aaRSs in each (Figure 1B and Table 3). This analysis presents a conundrum where none of the translational compartments in the three fungi seem independent for generating all 20 charged tRNAs. It is likely that some of the identified aaRSs have dual sub-cellular localizations within the cell. In addition, transport of charged tRNAs between cellular compartments could compensate for absence of any particular aaRS in a given chamber like cytoplasm or mitochondria, as in the case of other organisms [48].

**Table 1:** **ORFs in** ***A. niger*** **having aaRS or associated domains**

	aaRS domain	ORF	Predicted sub-cellular localization	Protein length	E-value for HMM prediction	Identity with human homolog (%)
Class I	ArgRS	197261-mRNA	Cyto	646	NA	36
	CysRS	211236-mRNA	Cyto	804	0	39
	GlnRS	45754-mRNA	Cyto	625	4.4e-247	44
	GluRS	56891-mRNA	Nucl	712	0	45
	GluRS	121468-mRNA	Mito	427	3.5e-155	37
	IleRS	52642-mRNA	Cyto	1077	0	53
	IleRS	212883-mRNA	Mito	1524	0	36
	LeuRS	183116-mRNA	Mito	989	0	38
	LeuRS	52554-mRNA	Cyto	1126	0	45
	MetRS	208481-mRNA	Cyto	659	0	48
	TrpRS	54362-mRNA	Cyto	433	6e-156	55
	TrpRS	209919-mRNA	Mito	391	6e-156	40
	TyrRS	54566-mRNA	Mito	611	5.1e-123	41
	ValRS	194077-mRNA	Cyto	1315	0	46
Class II	AlaRS	51231-mRNA	Mito	1019	7.4e-37	48
	AsnRS	210632-mRNA	Mito	543	1.5e-146	37
	AspRS	57039-mRNA	Mito	676	9.5e-179	38
	AspRS	56196-mRNA	Cyto	559	3.5e-281	48
	AsxRS	207470-mRNA	Cyto_nucl	574	4.7e-270	50
	AsxRS	55404-mRNA	Nucl	952	0	45
	GlyRS	57294-mRNA	Mito	738	0	51
	GlyRS	50342-mRNA	Cyto	667	0	47
	HisRS	51854-mRNA	Mito	619	1.4e-287	53
	LysRS	47873-mRNA	Cyto	604	0	57
	PheRS	55517-mRNA	Mito	514	3.8e-230	38
	PheRS α	56159-mRNA	Cyto	518	2.8e-254	47
	PheRS β	211951-mRNA	Cyto_nucl	600	0	47
	ProRS	210319-mRNA	Cyto	604	0	53
	SerRS	57312-mRNA	Cyto	474	5.2e-262	48
	ThrRS	51819-mRNA	Cyto	718	0	50
aaRS related proteins	AlaX	189390-mRNA	Cyto	271	1.4e-33	24
	DTD	56197-mRNA	Cyto_mito	191	NA	47
	P43 homolog	210303-mRNA	Cyto	445	3.1e-159	42
	Asn synthase	57091-mRNA	Mito	573	8.8e-57	34
	Asn synthase	186429-mRNA	Cyto	694	1.5e-39	27
	GatA	170115-mRNA	Extr	504	1.7e-159	39
	GatB	172989-mRNA	Mito	601	2.2e-138	33
	GatF	173432-mRNA	Mito	886	8.1e-12	NA

NA in e-value indicates fungal sequences that could be identified by HMM search and were retrieved from NCBI using keyword search.

**Table 2:** **ORFs in** ***C. albicans*** **having aaRS or associated domains**

	aaRS domain	ORF	Predicted sub-cellular localization	Protein length	E-value for HMM prediction	Identity with human homolog (%)
Class I	ArgRS	orf19.3341	Cyto	622	1.2e-104	38
	CysRS	orf19.4931	Mito	781	0	42
	GlnRS	orf19.7064	Cyto	799	0	38
	GluRS	orf19.2415	Mito	509	1.9e-199	35
	GluRS	orf19.7057	Cyto	725	NA	42
	IleRS	orf19.2138	Nucl	1088	0	53
	IleRS	orf19.2382	Mito	973	0	36
	LeuRS	orf19.2560	Cyto	1097	0	46
	LeuRS	orf19.5705	Cyto	861	0	37
	MetRS	orf19.3955	Cyto	748	0	51
	TrpRS	orf19.5226	Cyto	424	3e-235	56
	TrpRS	orf19.4299	Nucl	399	4.8e-156	42
	TyrRS	orf19.2694	Cyto	409	3.8e-170	53
	TyrRS	orf19.109	Mito	501	7.3e-152	34
	ValRS	orf19.1295	Mito	1119	0	48
Class II	AlaRS	orf19.5746	Cyto	969	0	50
	AsnRS	orf19.6698	Mito	489	6.2e-146	35
	AspRS	orf19.4478	Mito	698	5.5e-173	35
	AsxRS	orf19.2407	Cyto	578	0	58
	AsxRS	orf19.6702	Cyto	552	1.3e-261	52
	GlyRS	orf19.437	Nucl	652	0	50
	HisRS	orf19.4051	Mito	501	2.3e-271	52
	LysRS	orf19.6749	Nucl	594	0	56
	PheRS	orf19.2039	Mito	446	8.8e-218	39
	PheRS α	orf19.2960	Cyto	496	3.2e-244	47
	PheRS β	orf19.2573	Cyto_nucl	592	1.1e-284	45
	ProRS	orf19.6701	Cyto	691	0	57
	SerRS	orf19.269	Nucl	458	3.8e-245	46
	ThrRS	orf19.5685	Nucl	706	0	59
aaRS related proteins	DTD2	orf19.297	Cyto	163	NA	48
	AlaX	orf19.5239	Cyto_nucl	471	2.1e-127	31
	P43 homolog	orf19.2422	Cyto	369	1.9e-130	53
	Asn synthase	orf19.3626	Nucl	704	1e-178	25
	Asn synthase	orf19.198	Cyto	573	1.1e-58	35
	GatA	orf19.3956	Mito	450	1.7e-135	34
	GatB	orf19.2494	Nucl	488	9.7e-141	32
	GatF	orf19.4092	Nucl	165	NA	NA

NA in e-value indicates fungal sequences that could be identified by HMM search and were retrieved from NCBI using keyword search.

**Table 3:** **ORFs in** ***C. neoformans*** **having aaRS or associated domains**

	aaRS domain	ORF	Predicted sub-cellular localization	Protein length	E-value for HMM prediction	Identity with human homolog (%)
Class I	ArgRS	CNBG3480	Cyto	622	NA	37
	CysRS	CNBB4810	Cyto	785	0	42
	GlnRS	CNBA0720	Cyto	858	5.2e-300	40
	GluRS	CNBK2470	Mito	588	2.3e-197	35
	GluRS	CNBF4640	Cyto	728	3.1e-295	43
	IleRS	CNBA2140	Cyto_nucl	1094	0	51
	IleRS	CNBN1610	Mito	1032	0	36
	LeuRS	CNBM1150	Cyto	1114	0	46
	MetRS	CNBD2070	Cyto	724	0	46
	TrpRS	CNBJ3070	Mito	641	5.5e-221	55
	TyrRS	CNBA1270	Cyto	819	3.3e-218	47
	TyrRS	CNBJ0260	Mito	478	1.2e-169	47
	ValRS	CNBB3840	Mito	1109	0	47
Class II	AlaRS	CNBF2180	Mito	1012	0	49
	AsnRS	CNBD2410	Mito	513	1.7e-157	38
	AsnRS	CNBE1330	Cyto	600	1.8e-251	48
	AspRS	CNBK1200	Mito	693	8.5e-193	36
	AsxRS	CNBG4380	Cyto	884	0	42
	GlyRS	CNBF3810	Cyto	673	0	46
	HisRS	CNBC0810	Mito	586	4.2e-270	47
	LysRS	CNBH3030	Mito	630	0	52
	PheRS	CNBC0600	Mito	476	4.5e-214	40
	PheRS α	CNBJ1210	Cyto	510	1.1e-250	51
	PheRS β	CNBC3360	Nucl	632	4.1e-283	44
	ProRS	CNBB0150	Mito	740	0	55
	SerRS	CNBD0870	Cyto	461	2.6e-225	40
	ThrRS	CNBB5220	Mito	772	0	56
aaRS related proteins	AlaX	CNBA4150	Cyto	523	1.1e-108	29
	DTD	CNBB3530	Cyto	234	1e-41	47
	P43 homolog	CNBF3180	Cyto	383	1.8e-124	47
	Asn synthase	CNBK3010	Mito	572	1.7e-196	34
	Asn synthase	CNBL0870	Mito	592	1.6e-65	34
	GatA	CNBD1400	Mito	493	1.7e-119	33
	GatB	CNBG4210	Mito	521	1.7e-176	37

NA in e-value indicates fungal sequences that could be identified by HMM search and were retrieved from NCBI using keyword search.

We also sought to catalog trans-editing domains within these fungal genomes. D-Tyrosyl-tRNATyr deacylase (DTD) catalyzes hydrolysis of bond between D-amino-acid and tRNA. These enzymes play an important role in evading toxicity resulting from accumulations of D-amino acids within the cell [49]. Trans-editing domains such as AlaX maintain fidelity of tRNA charging by hydrolyzing incorrectly charged amino acid attached to tRNA^Ala^ [50]. Hence, in addition to profile HMM search for 20 canonical aaRSs; screening for aaRS-associated proteins was also performed (see Methods section for details). Our results suggest that trans-editing domains such as DTD and AlaX are found in all the three fungi (Tables 1, 2 and 3). Similarly, the possibility of alternate route for Gln-tRNA^Gln^ and Asn-tRNA^Asn^ synthesis was examined [33, 51]. In this alternate mechanism, a non-discriminating aaRS catalyze the synthesis of Glu-tRNA^Gln^ or Asp-tRNA^Asn^ followed by catalysis by a corresponding amidotransferase to generate Gln-tRNA^Gln^ or Asn-tRNA^Asn^ respectively. Curiously, two separate proteins corresponding to amidotransferase (Asn synthase) were observed in all the three fungal genomes (Tables 1, 2 and 3). The existence of Asn synthase corroborates with the presence of above identified non-discriminating domains (AsxRS) within the host fungi (Tables 1, 2 and 3). Similar to Asn synthase, Gln synthase is a transamidase that catalyzes the synthesis of Gln-tRNA^Gln^ from Glu-tRNA^Gln^. In mammals, heterotrimeric Gln synthase (GatABC) is frequently observed within mitochondria for synthesis of Gln-tRNA^Gln^ [52]. In addition to aminoacylation activity, subunit proteins of Gln synthase have been shown to be important for proper functioning of mitochondrial activities unrelated to protein translation [52]. We additionally identified homologs for subunit A and B of Gln synthase in the three fungi (Tables 1, 2 and 3), however, homologs for GatF subunit could be ascertained only in case of *A. niger* and *C. albicans*.

In some eukaryotes, a few aaRSs associate with each other and with auxillary proteins (called P18, P38, and P43) to form multi-synthetase complex (MSC) [53, 54]. It has been proposed that MSC improves translational efficiency by channeling tRNAs to aaRSs and ribosomes [55]. In addition, MSC may serve as a reservoir of pro-cytokines and other regulatory molecules that can be released per physiological requirements [56]. A central component of MSC is P43, a polypeptide of ~43 kDa that harbors tRNA binding domain. P43 is associated with multitude of cellular functions such as protein synthesis, axonal development, glucagon secretion, and autoimmune suppression [57]. Our profile HMM-based screening revealed existence of P43 variants in all the three fungal genomes (Tables 1, 2 and 3). Presence of MSC and its constituents now remain to be addressed experimentally.

### Domain architecture for aaRSs in *A. niger*

**Figure 2:**
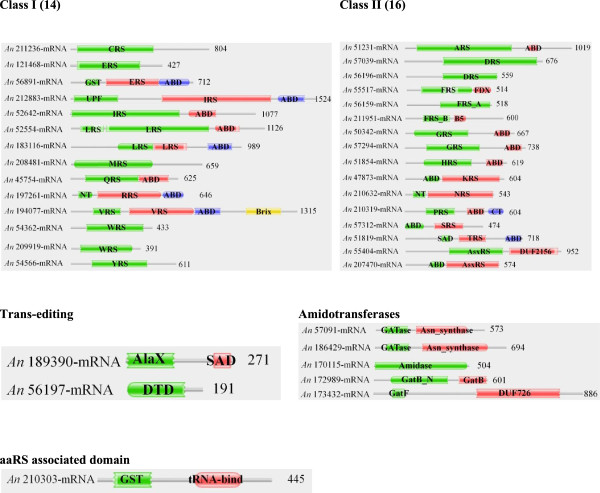
**Domain annotations for predicted aminoacyl-tRNA synthetases (aaRSs) in** ***A. niger*** **.** Catalytic domains are labeled with three letter code for corresponding aaRS and number at the end of sequence denotes the length of the protein. ABD - Anti-codon binding, FDX - Ferridoxin-fold ABD, UPF - Unknown protein family, GST - Glutathione-S-transferase, NT - N-terminal, CT - C-terminal, DUF2156 - Domain of unknown function, SAD - Second associated domain.

**Figure 3:**
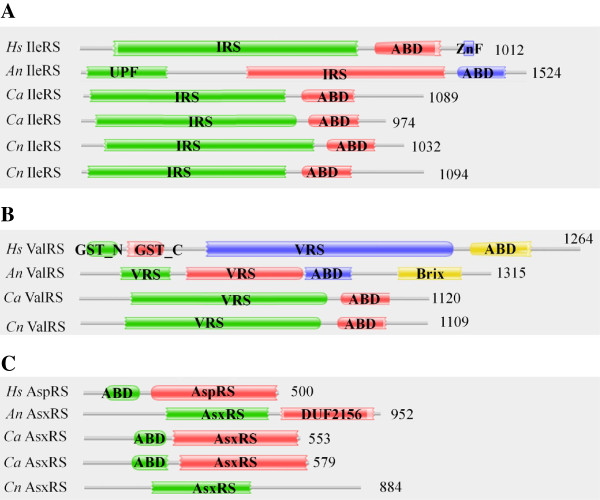
**Comparison of domain architectures of** ***A. niger*** **and** ***H. sapiens*** **aaRSs.** Homologous proteins from *C. albicans* and *C. neoformans* are also shown. **(A)** An uncharacterized protein family (UPF) domain at the N-terminal of *A. niger* IleRS (212883-mRNA) is absent in the homolog from *H. sapiens. A. niger* and *H. sapiens* IleRSs share ~36% sequence identity. **(B)** Comparison of domain architectures of ValRS from *A. niger* (194077-mRNA) and *H. sapien*s. Brix domain at the C-terminal is unique to *A. niger* ValRS, and these two ValRSs are ~46% identical. **(C)** *A. niger* AsxRS has a unique domain of unknown function (DUF) C-terminal to the catalytic domain. *A. niger* AsxRS and *H. sapiens* AspRS are ~45% identical.

Profile HMM searches identified a total of 29 aaRS in *A. niger* – 14 belonging to class I and 15 to class II. Among class I aaRSs – IleRS, LeuRS, GlnRS, ArgRS, and MetRS had N-terminal catalytic domain followed by anticodon binding domain (ABD) towards the C-terminal (Figure 2). Two different proteins corresponding to IleRS were predicted to localize in cytoplasm (52642-mRNA) and extracellular space (212883-mRNA) respectively. However, the synthetase domain of IleRS (212883-mRNA) shares ~36% sequence identity with human mitochondrial IleRS and therefore its sub-cellular localization is likely to be mitochondrial rather than extracellular. Interestingly, one IleRS (212883-mRNA) contains an addition uncharacterized protein family (UPF0183) domain at its N-terminal (Figure 2). BLAST searches for this IleRS (212883-mRNA) against human genome failed to identify a homolog for the UPF part of the sequence (Figure 3A). Two separate proteins for LeuRS were identified, putatively localized to cytoplasm (52554-mRNA) and mitochondria (183116-mRNA). Mitochondrial (121468-mRNA) and potentially nuclear (565891-mRNA) copies of GluRS were also identified. In latter, an additional glutathione-S-transferase C-terminal domain (GST_C, an α-helix containing structural domain) is evident at the N-terminus. Human bifunctional GluRS/ProRS also contains GST_C domain at the N-terminus and these proteins shares 45% identity with *A. niger* GluRS. The GST_C domain fusion is also present in human MetRS and ValRS. Curiously, the GlnRS (45754-mRNA) lacks N-terminal RNA binding region that is present in human and other fungal homologs (Figure 2). The TrpRSs are two-copy enzymes in *A. niger*, one each for cytoplasm (54362-mRNA) and mitochondria (209919-mRNA). In case of CysRS, GluRS, MetRS, TrpRS and TyrRS the Pfam server could only annotate their catalytic domains within these sequences. However, based on the catalytic domain positioning it appears likely that their anti-codon binding domains lie towards the C-termini (Figure 2).

Surprisingly, *A. niger* ValRS (194077-mRNA) displays an additional Brix domain at its C-terminus (Figure 2). Protein families containing Brix domain participate in ribosomal biogenesis and rRNA binding [58]. These observations raise an intriguing possibility for cytoplasmic ValRS (194077-mRNA) to participate in rRNA production. The Brix domain in ValRS (194077-mRNA) appears to be unique to *A. niger* and is not observed in Human ValRSs (Figure 3B). In order to manually annotate the gene for *A. niger* VRS (having Brix domain fusion), BLAST searches for 194077-mRNA were performed against the *A. niger* transcriptome database located at the Broad Institute, USA. A highly significant hit was obtained spanning the fungal full-length protein sequence (Additional file 1: Figure S2A). These results indicate that chromosome 16 within *A. niger* genome codes for VRS-Brix domain fusion protein. Structural modeling for the Brix domain from 194077-mRNA confirms conservation in the catalytic fold with respect to its homologs (Additional file 1: Figure S2B).

The class II set of aaRSs in *A. niger* showed heterogeneity in the relative organization of catalytic and anti-codon binding (ABD) domains. AlaRS, PheRS, GlyRS, HisRS, ProRS and ThrRS had catalytic domains at the N-termini of anti-codon binding domain (Figure 2). While in case of LysRS, SerRS and AsxRS the catalytic domain is present at the C-terminal of the anti-codon binding domain (Figure 2). AspRSs (56196-mRNA and 57039-mRNA) were the only class II proteins in *A. niger* for which anti-codon binding domain could not be annotated. However, based on Pfam assignment of catalytic domain in these proteins it appears likely that the ABD lies at the N-terminal region (Figure 2). In case of PheRS, two proteins were found with different predicted sub-cellular localization. Cytoplasmic PheRS was predicted to be a heterodimer comprising of α (56159-mRNA) and β (211951-mRNA) subunits. On the other hand, mitochondrial PheRS (55517-mRNA) retain a ferrodoxin fold containing anti-codon binding domain (Figure 2). These observations suggest that the quaternary (tetrameric) structure of mitochondrial PherRS (55517-mRNA) comprises only of one protomer while cytoplasmic PheRS has αβ heterodimer as the basic subunit of the tetramer.

Interestingly, we identified two non-discriminating aspartyl-tRNA synthetases (AsxRS) that were predicted to localize in cytoplasm (207470-mRNA) and nucleus (55404-mRNA) respectively. These enzymes catalyze the charging of both tRNA^Asp^ and tRNA^Asn^ with aspartate [51]. Asp-tRNA^Asn^ thus generated is further acted upon by amidotransferase leading to synthesis of Asn-tRNA^Asn^ [59]. Curiously, one AsxRS (55404-mRNA), predicted to have nuclear location, has an additional domain of unknown function (DUF) at the C-terminal (Figure 2) which is missing from the human homolog (Figure 3C). Profile HMM searches for the amidotransferases identified two Asn synthase – 57091-mRNA (mitochondrial) and 186429-mRNA (cytoplasmic) (Figure 2). These amidotranferases together with non-discriminating aspartyl-tRNA synthetase (AsxRS) likely participate in the indirect synthesis of Asn-tRNA^Asn^. Our results show that there is only one AsnRS which is localized in mitochondria. Therefore it appears that the mitochondrial repertoire of Asn-tRNA^Asn^ is maintained by AsnRS as well as by the combined activity of AsxRS and Asn synthase. The cytoplasmic pool of Asn-tRNA^Asn^ is likely maintained only by the collective activity of AsxRS and Asn synthase enzymes. Homologs for Gln synthase subunits A (170115-mRNA), B (172989-mRNA) and F (173432-mRNA) were also identified (Table 1). These three subunits together constitute an active GatFAB enzyme within the mitochondria and facilitate the synthesis of Gln-tRNA^Gln^ within the organelle. Curiously, GatF (173432-mRNA) has an additional domain (DUF726) at its C-terminal (Figure 2). It is possible that the mitochondrial GluRS, like its human homolog, is essentially a non-discriminating enzyme that synthesizes Glu-tRNA^Gln^ which acts as a substrate for heterodimeric Gln synthase to yield Gln-tRNA^Gln^.

**Figure 4:**
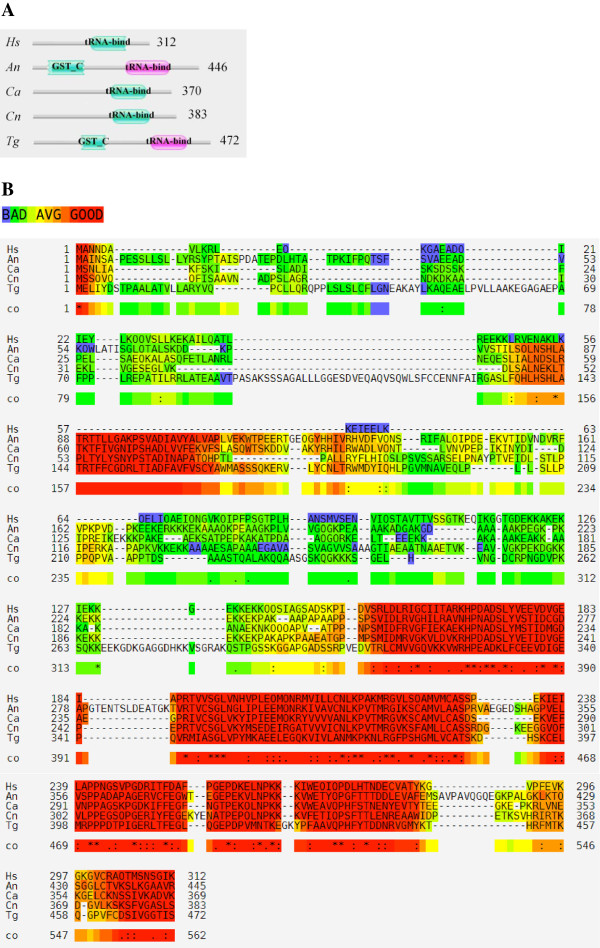
**Comparison of P43 homologs from different genomes. (A)** Pfam-based domain organization for P43 homologs from *Homo sapiens* (*Hs*), *A. niger* (*An*), *C. albicans* (*Ca*), *C. neoformans* (Cn), and *Toxoplasma gondii* (*Tg*). **(B)** Sequence alignment for P43s with color codes showing sequence conservation – low (blue) to high (red). Overall, there is high conservation in the tRNA-binding domain at the C-terminal and poor homology at the N-terminals.

Significant matches for standalone trans-editing and deacylase domains were also observed for two proteins – 189390-mRNA and 56197-mRNA that contains AlaX and DTD domains respectively. We also identified a P43 homolog (210303-mRNA) in *A. niger*. Interestingly, *A. niger* P43 has GST C-terminal domain at the N-terminal of the tRNA binding region, which is different from the human counterpart. Indeed, GST_C domain is absent in human P43, however, it is present in P43 homolog from *Toxoplasma gondii* [17] (Figure 4A). Multiple sequence alignment for P43 homologs shows high conservation in the tRNA binding domains between human, the three fungi, and *T. gondii* P43s (Figure 4B). However, *A. niger* P43 sequence has two insertions (one of 4 residues and another of 9 residues) within the structurally conserved tRNA binding domain. Further, the N-termini of these proteins show poor homology amongst each other. Finally, GluRS (56891-mRNA), HisRS (51854-mRNA), and LeuRS (52554-mRNA) were observed to have ‘ELR’ motifs in their N-terminal regions (Additional file 1: Table S2). In human and other TyrRSs this motif can impart cytokine-related activities [21, 60–62] which are untested in the three fungi so far. Mapping of ELR motifs onto the modeled structures showed that the ELR motif appeared in surface exposed loop regions in these three aaRSs (data not shown).

### Domain architecture for aaRSs in *C. albicans*

**Figure 5:**
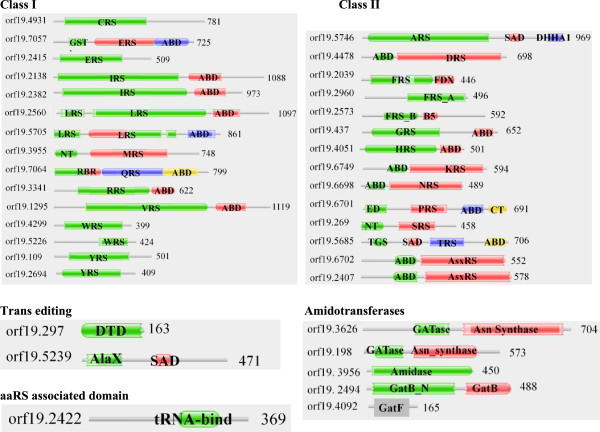
**Domain annotations for predicted aminoacyl-tRNA synthetases (aaRSs) in** ***C. albicans*** **.** Catalytic domains are labeled with three letter code for corresponding aaRS and number at the end of the sequence denotes the length of the protein. ED - Editing domain, TGS – (ThrRS, GTPase, and SpoT) domain, RBR – non-specific RNA binding region, DHHA1 - DHH associated domain, and other domains are same as in Figure 2.

The total number of aaRSs identified in *C. albicans* was 28 with 15 and 13 members in class I and class II respectively. The general organization of domains amongst class I aaRSs was prototypical: catalytic domain followed by anticodon binding domain (Figure 5). For ~50% of class I aaRSs, two different proteins were identified specific for a particular amino acid – presumably one aaRS each for cytoplasm and mitochondria (Figure 5). Class I aaRSs for which only one copy was identified includes CysRS (orf19.4931), MetRS (orf19.3955), GlnRS (orf19.7064), ArgRS (orf19.3341), and ValRS (orf19.1295). The cytoplasmic copy of GluRS (orf19.7057) had an additional glutathione-S-transferase (GST_C) domain appended at the N-terminus (Figure 5), like the human bifunctional glutamate/proline--tRNA synthetase. In case of MetRS (orf19.3955), an additional N-terminal domain was identified based on Pfam annotation. This domain is again unique to *C. albicans* MetRS and is absent from the other two fungal MetRSs analyzed here as well as from the human MetRS (Figure 5). Two proteins each for IleRS (orf19.2138 and orf19.2382) and LeuRS (orf19.2560 and 5705) were predicted to have anti-codon binding domain at the C-terminal of catalytic domain (Figure 5). Two differentially localized proteins each for TrpRS (orf19.4299 and orf19.5226) and TyrRS (orf19.109 and orf19.2694) were also identified (Table 2 and Figure 5).

Class II aaRSs were generally present as a single protein except for PheRS and AsxRS. The two PheRSs had different predicted sub-cellular localization with one likely in cytoplasm and other in mitochondria. Like the *A. niger* mitochondrial PheRS, *C. albicans* homolog (orf19.2039) also has anti-codon binding domain at the C-terminal of catalytic domain. Further, cytoplasmic PheRS prototypically encompass α (orf19.2960) and β (orf19.2573) subunits (Figure 5). The AlaRS (orf19.5746) has second associated domain (SAD) and DHHA1 domains C-terminal to its catalytic domain (Figure 5). These two domains are characteristic of AlaRSs and contribute to the catalytic activity of the enzyme [63]. ProRS (orf19.6701), in addition to catalytic and anti-codon binding domains, had two additional domains one at its termini: N-terminal (editing domain) and a C-terminal domain. ThrRS (orf19.5685) had TGS (ThrRS, GTPase, and SpoT) and SAD (second additional domain) domains present N-terminal to catalytic domain (Figure 5). Two separate non-discriminating aspartyl-tRNA synthetase (AsxRS) were also identified – orf19.6702 and orf19.2407 (Figure 5).

Amongst aaRS-associated proteins, two Asn synthases were identified in *C. albicans* – orf19.3626 (predicted nuclear) and orf19.198 (predicted cytoplasm). These two enzymes could act in tandem with two AsxRSs to generate Asn-tRNA^Asn^ in an indirect manner. In addition, subunit components of Gln synthase were also identified – GatA (orf19.3956), GatB (orf19.2494) and GatF (orf19.4092) (Table 2 and Figure 5). *C. albicans* mitochondrial GluRS (orf19.2415) shares ~35% identity with human mitochondrial GluRS (which is non-discriminating), raising the possibility of indirect synthesis of Gln-tRNA^Gln^ by the action of Gln synthase on Glu-tRNA^Gln^. Standalone trans-editing domains like AlaX (orf19.5239) and DTD (orf19.297) were also identified (Figure 5). Our results confirm the presence of P43 homolog (orf19.2422) within the *C. albicans* genome (Figure 5). Three aaRSs – GluRS, HisRS, and LysRS in *C. albicans* have ‘ELR’ motif at the N-terminal region (Additional file 1: Table S2). The ELR motifs in these three aaRSs were surface exposed in modeled three-dimensional structures (data not shown).

### Domain architecture for aaRSs in *C. neoformans*

**Figure 6:**
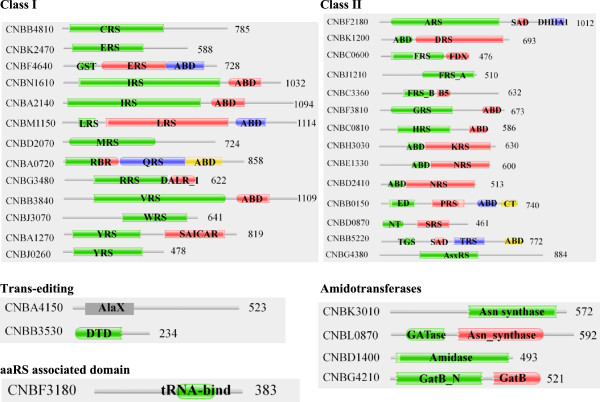
**Domain annotations for predicted aminoacyl-tRNA synthetases (aaRSs) in** ***C. neoformans*** **.** The catalytic domains are labeled with three letter code for corresponding aaRS and number at the end of the sequence denotes the length of the protein. DALR_1 - (Asp, Ala, Leu, and Arg) domain, SAICAR - Phosphoribosylaminoimidazolesuccinocarboxamide domain. Other domains are same as in Figure 2.



**Figure 7:**
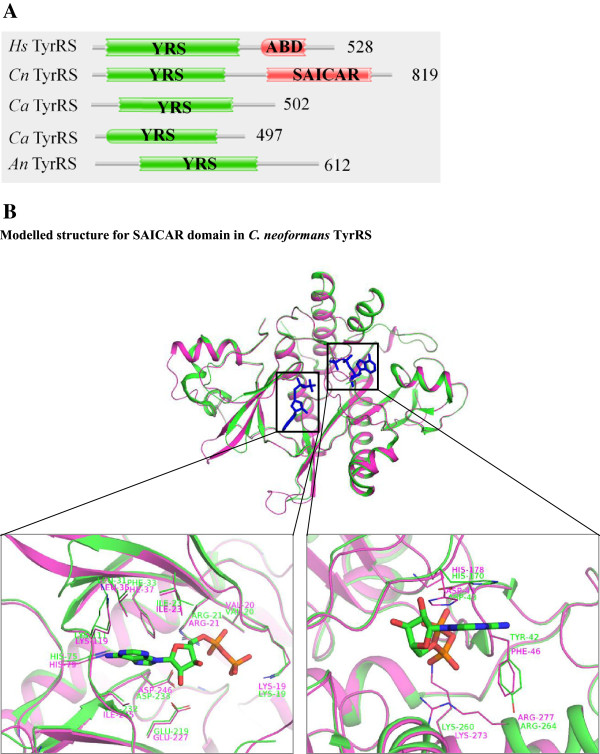
**Sequence and structural analyses for tyrosyl-tRNA synthetase from** ***C. neoformans.*** **(A)** Comparison of domain architectures for *C. neoformans* TyrRS (CNBA1270) and *H. sapiens* cytoplasmic TyrRS based on the Pfam domain assignment. SAICAR domain at the C-terminal of *C. neoformans* TyrRS is absent in the homolog from *H. sapiens* and these two TyrRSs are ~47% identical in the TyrRS catalytic domain region. **(B)** Superimposition of modeled *C. neoformans* SAICAR domain in TyrRS (magenta) and crystal structure of SAICAR synthase (PDB ID 2CNQ) from *Saccharomyces cerevisiae* (green) with adenosine-di-phosphate (ADP, blue). The two proteins share 51% sequence identity. Inset shows sequence and structural conservation of ADP interacting residue in the two proteins.

Profile HMM-based searches identified 26 aaRSs with 13 each belonging to class I or II (Figure 6). Amongst class II aaRSs, two aaRSs were observed only for PheRS and GlnRS. The gene structures for other class II aaRSs were mostly similar to those described for *A. niger* and *C. albicans*. Two encoded proteins were observed for IleRS (CNBN1610 and CNBA2140) and TyrRS (CNBA1270 and CNBJ0260) (Table 3). Intriguingly, in one of the TyrRSs (CNBA1270), a SAICAR domain (a protein module that synthesizes 4-(N-succinylcarboxamide)-5-aminoimidazole ribonucleotide) was identified towards the C-terminal in fusion with the prototypical TyrRS. Predicted sub-cellular localization suggests this unusual fusion TyrRS (CNBA1270) to be cytoplasmic. Other SAICAR domain containing proteins (such as phospho-ribosyl-amino-imidazole-succino-carboxamide synthase) are involved in *de novo* purine biosynthetic pathway and catalyze the following reaction [64]:

Vertebrate SAICAR synthetases are significantly different from their microbial homologs both in terms of subunit structure (vertebrate SAICAR synthetase are multimeric proteins) and functions (vertebrates have bifunctional enzymes having AIR carboxylase and SAICAR synthetase activity [65]). A recent study has suggested microbial SAICAR synthetases as potential drug target [66]. Significantly, we could not identify a homolog for TyrRS-SAICAR fusion protein (CNBA1270) in humans and hence these unusual fused domains seems unique to *C. neoformans* (Figure 7A). Intriguingly, BLAST searches against non-redundant databases revealed that homologs for TyrRS-SAICAR fusion protein exist only in some other fungi such as *Cryptococcus gatti*, *Trichosporon asahii* and *Tremella mesenterica*. In order to validate this domain fusion in the case of *C. neoformans*, BLAST searches for CNBA1270 were done against transcriptome database available at BROAD institute, USA. A highly significant transcript was obtained corresponding to the computationally predicted protein sequence (Additional file 1: Figure S3). These results reinforce presence of a chromosomal region in *C. neoformans* genome that encodes for YRS-SAICAR fusion protein. Modeling of the tertiary structure for SAICAR domain in CNBA1270 confirmed conservation of key residues within the ATP binding pocket (Figure 7B). These observations further substantiate the hypothesis that this fusion domain protein could be potentially targeted for therapeutic development against *C. neoformans*.

**Figure 8:**
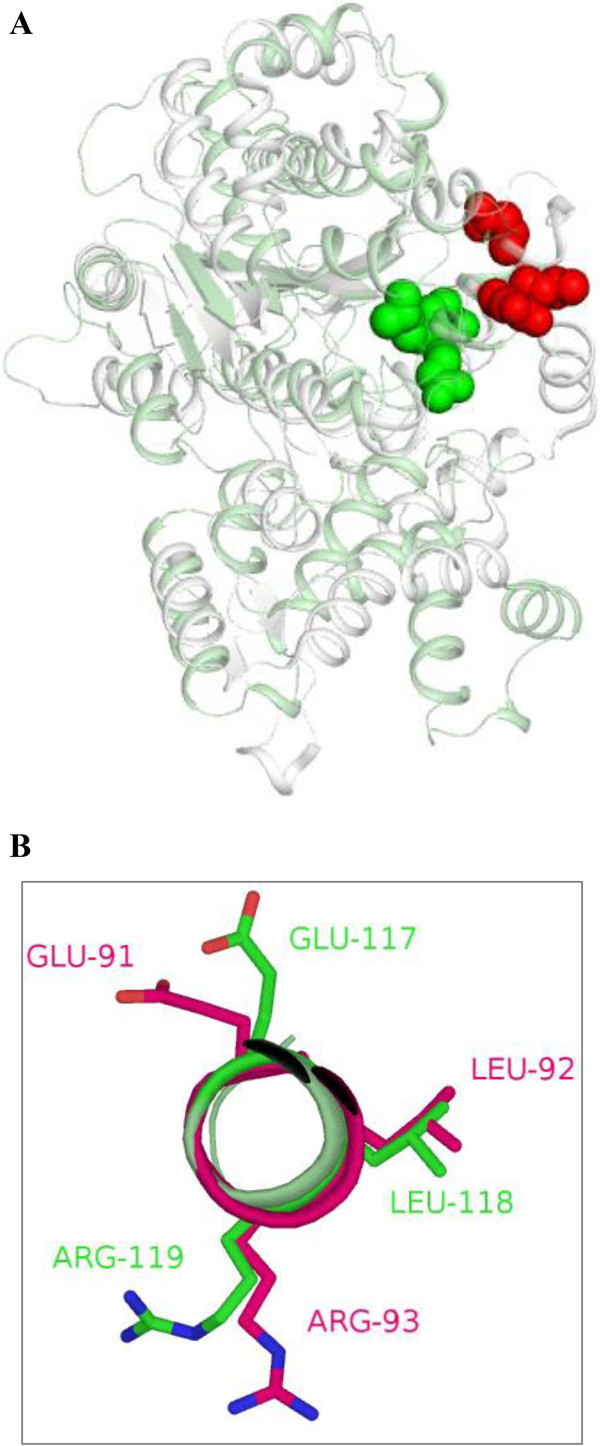
**Superimposition for crystal structures of human TyrRS (white, PDBID 1N3L) and modeled structure of** ***C. neoformans*** **TyrRS (light green). (A)** Residues constituting ELR motif in the two proteins are shown as spheres in red and green for human and *C. neoformans,* respectively. **(B)** Superimposition of the ELR motifs from the two proteins.

Among aaRS related proteins, four polypeptides were predicted to have amidotransferases activity – two Asn synthases (CNBK3010 and CNBL0870) and other two Gln synthases (subunit A (CNBD1400) and subunit B (CNBG4210)). We were not able to identify any homolog for GatF subunit. Interestingly, all these four amidotranferases were predicted to localize within the mitochondria (Table 3). In addition, a standalone trans-editing AlaX (CNBA4150) and DTD (CNBB3530) were also identified (Figure 6). Interestingly, the mitochondrial TyrRS-SAICAR fusion protein (CNBJ0260) had an ‘ELR’ motif at its N-terminal (Additional file 1: Table S2). This motif in human TyrRS is responsible for cytokine activity. Structural modeling for *C. neoformans* TyrRS part of the fusion protein suggests that the ‘ELR’ motif is likely to be solvent accessible (Figure 8A). Further, the ‘ELR’ motifs in human and *C. neoformans* TyrRSs are part of α helices that superimpose well (Figure 8B). Presence of ELR motif at the N-terminal and an additional unique SAICAR domain towards C-terminal makes *C. neoformans* mitochondrial TyrRS (CNBJ0260) an interesting target for further investigation. In addition, we noted that ProRS (CNBB0150) also has an ‘ELR’ motif towards its N-terminal (Additional file 1: Table S2), which when modeled indicated surface exposure for this motif.

## Conclusions

Treatment and prevention of opportunistic fungal infections in humans presents an emerging challenge. *Aspergillus, Candida* and *Cryptococcus* are three highly pathogenic fungi, particularly in immuno-compromised patients. Dissecting and understanding critical fungal protein machineries is therefore vital for establishing a base for launching new therapeutics against these pathogens. Within microbial proteomes, aminoacyl-tRNA synthetases have already been exploited for drug discovery [67]. Specifically for fungal aaRS, a novel boron-containing molecule 5-fluoro-1,3-dihydro-1-hydroxy-2,1-benzoxaborole (AN2690) that inhibits yeast cytoplasmic leucyl-tRNA synthetase by trapping enzyme-bound tRNA^Leu^ in editing conformation is now an anti-fungal agent in the market [22]. Hence, proof-of-principle exists that can now be expanded to target a much larger number of fungal aaRSs. Our profile HMM-based identification and annotation confirms the existence of all 20 aaRSs in the three studied fungal genomes. In addition, fungal DTDs, AlaXs, P43s and amidotransferases have also been comprehensively annotated. Putative distribution of fungal aaRSs in different sub-cellular compartments within the fungi provides a base for experimental validation. Our results also highlight fungal processes that enable multiple localization of aaRSs and/or of charged tRNAs between cytoplasm and mitochondria in these three fungi since neither their cytoplasm nor mitochondria individually harbor a complete set of 20 aaRSs.

Overall, the three fungal genomes harbor similar number of aaRSs. In *A. niger*, *C. albicans*, and *C. neoformans* there exist 8, 7, and 5 aaRSs that are encoded by two different set of genes, presumably for mitochondrial localization (Figure 1A). PheRS, GluRS, and IleRS are the three aaRSs that are two-gene sets in all the three fungal genomes. The distribution for aaRS associated proteins is conserved among the three studies fungi. Our results predict that *C. neoformans* Gln synthase (Gat) maybe heterodimeric unlike its homologs in other two fungi where it is likely to be heterotrimeric. None of the aaRS in *C. neoformans* were predicted to localize within nucleus while 2 and 6 aaRSs in *A. niger* and *C. albicans* were predicted to have nuclear localization (Figure 1B).

The aaRSs identified in these fungal genomes share some similarities with their human homologs (Tables 1, 2 and 3) but also many critical differences (Figures 3 and 6). *A. niger* IleRS and AsxRS have unique domain fusions which are absent in homologs from the other two fungi and from humans (Figure 3). ValRS and TyrRS from *A. niger* and *C. neoformans* also harbor novel functionally-characterized domain fusions which are absent from humans (Figures 3 and 7). Further structural and functional characterization of the unique and novel fungal aaRSs is required before they can be exploited for development of anti-fungal compounds. In addition, experimental analyses of fungal aaRSs that display unusual domain fusions may uncover their non-canonical functions. We found N-terminal GST_C domains in *A. niger* P43 which is distinct feature for this protein in comparison to other two fungi and humans (Figure 4). Our results also reveal presence of novel and unique fusion proteins where domains such as DUF2156 and SAICAR are appended to the aaRS structural cores (Figures 2 and 6).

In summary, the comprehensive genomic cataloging of aaRSs from pathogenic fungi detailed here warrants further experimental validation and exploration. The results presented here provide insights into protein translation enzymes within these pathogenic fungi that can be targeted for developing new drugs against these microbes.

## Availability of supporting data

The data sets supporting the results of this article are included within the article and Additional file 1.

## Electronic supplementary material

Additional file 1: Table S1 and Table S2: Having the details of the dataset used for analyses and summary of fungal aaRSs with ELR motif, respectively. **Figure S1.** Shows details for signal sequences identified in fungal aaRSs. **Figure S2.** Shows (A) schematic representation for genome and transcriptome sequencing analyses for VRS-Brix and (B) modelling for Brix domain from *C. neoformans*. **Figure S3.** Schematic representation for genome and transcriptome sequencing analyses for YRS-SAICAR fusion protein in *C. neoformans*. (DOCX 356 KB)
