# Supplementary material for: Novel and unique domains in aminoacyl-tRNA synthetases from human fungal pathogens Aspergillus niger, Candida albicans and Cryptococcus neoformans
Source: BMC Genomics. 2014 Dec 5;15(1):1069. doi: 10.1186/1471-2164-15-1069 (PMC4301749; doi:10.1186/1471-2164-15-1069)

**Table S1:** List of proteins used for building profile HMMs.

| **UniProt ID** | **Protein names (Enzyme classification)** | **Length** |
| --- | --- | --- |
| Q9BTE6 | Alanyl-tRNA editing protein Aarsd1 | 412 |
| Q12904 | Aminoacyl tRNA synthase complex-interacting multifunctional protein 1 ( p43) | 312 |
| Q9NWL6 | Asparagine synthetase domain-containing protein 1 | 643 |
| Q8TEA8 | D-tyrosyl-tRNA(Tyr) deacylase 1 (EC 3.1.-.-) | 209 |
| Q96FN9 | Probable D-tyrosyl-tRNA(Tyr) deacylase 2 (EC 3.1.-.-) | 168 |
| Q9H0R6 | Glutamyl-tRNA(Gln) amidotransferase subunit A, mitochondrial (EC 6.3.5.7) | 528 |
| O75879 | Glutamyl-tRNA(Gln) amidotransferase subunit B, mitochondrial (EC 6.3.5.-) | 557 |
| O43716 | Glutamyl-tRNA(Gln) amidotransferase subunit C, mitochondrial (EC 6.3.5.-) | 136 |
| Q9HD40 | O-phosphoseryl-tRNA(Sec) selenium transferase (EC 2.9.1.2) | 501 |
| P49588 | Alanine--tRNA ligase, cytoplasmic (EC 6.1.1.7) | 968 |
| Q5JTZ9 | Alanine--tRNA ligase, mitochondrial (EC 6.1.1.7) | 985 |
| P49589 | Cysteine--tRNA ligase, cytoplasmic (EC 6.1.1.16) | 748 |
| Q9HA77 | Probable cysteine--tRNA ligase, mitochondrial (EC 6.1.1.16) | 564 |
| P14868 | Aspartate--tRNA ligase, cytoplasmic (EC 6.1.1.12) | 501 |
| Q6PI48 | Aspartate--tRNA ligase, mitochondrial (EC 6.1.1.12) | 645 |
| Q5JPH6 | Probable glutamate--tRNA ligase, mitochondrial (EC 6.1.1.17) | 523 |
| P07814 | Bifunctional glutamate/proline--tRNA ligase | 1512 |
| Q9Y285 | Phenylalanine--tRNA ligase alpha subunit (EC 6.1.1.20) | 508 |
| Q9NSD9 | Phenylalanine--tRNA ligase beta subunit (EC 6.1.1.20) | 589 |
| O95363 | Phenylalanine--tRNA ligase, mitochondrial (EC 6.1.1.20) | 451 |
| P41250 | Glycine--tRNA ligase (EC 6.1.1.14) | 739 |
| P12081 | Histidine--tRNA ligase, cytoplasmic (EC 6.1.1.21) | 509 |
| P49590 | Probable histidine--tRNA ligase, mitochondrial (EC 6.1.1.21) | 506 |
| P41252 | Isoleucine--tRNA ligase, cytoplasmic (EC 6.1.1.5) | 1262 |
| Q9NSE4 | Isoleucine--tRNA ligase, mitochondrial (EC 6.1.1.5) | 1012 |
| Q15046 | Lysine--tRNA ligase (EC 6.1.1.6) | 597 |
| Q9P2J5 | Leucine--tRNA ligase, cytoplasmic (EC 6.1.1.4) | 1176 |
| Q15031 | Probable leucine--tRNA ligase, mitochondrial (EC 6.1.1.4) | 903 |
| P56192 | Methionine--tRNA ligase, cytoplasmic (EC 6.1.1.10) | 900 |
| Q96GW9 | Methionine--tRNA ligase, mitochondrial (EC 6.1.1.10) | 593 |
| O43776 | Asparagine--tRNA ligase, cytoplasmic (EC 6.1.1.22) | 548 |
| Q96I59 | Probable asparagine--tRNA ligase, mitochondrial (EC 6.1.1.22) | 477 |
| Q7L3T8 | Probable proline--tRNA ligase, mitochondrial (EC 6.1.1.15) | 475 |
| P47897 | Glutamine--tRNA ligase (EC 6.1.1.18) | 775 |
| P54136 | Arginine--tRNA ligase, cytoplasmic (EC 6.1.1.19) | 660 |
| Q5T160 | Probable arginine--tRNA ligase, mitochondrial (EC 6.1.1.19) | 578 |
| P49591 | Serine--tRNA ligase, cytoplasmic (EC 6.1.1.11) | 514 |
| Q9NP81 | Serine--tRNA ligase, mitochondrial (EC 6.1.1.11) | 518 |
| A2RTX5 | Probable threonine--tRNA ligase 2, cytoplasmic (EC 6.1.1.3) | 802 |
| P26639 | Threonine--tRNA ligase, cytoplasmic (EC 6.1.1.3) | 723 |
| Q9BW92 | Threonine--tRNA ligase, mitochondrial (EC 6.1.1.3) | 718 |
| P26640 | Valine--tRNA ligase (EC 6.1.1.9) | 1264 |
| Q5ST30 | Valine--tRNA ligase, mitochondrial (EC 6.1.1.9) | 1063 |
| P23381 | Tryptophan--tRNA ligase, cytoplasmic (EC 6.1.1.2) | 471 |
| Q9UGM6 | Tryptophan--tRNA ligase, mitochondrial (EC 6.1.1.2) | 360 |
| P54577 | Tyrosine--tRNA ligase, cytoplasmic (EC 6.1.1.1) | 528 |
| Q9Y2Z4 | Tyrosine--tRNA ligase, mitochondrial (EC 6.1.1.1) | 477 |
| P0AAR3 | Cys-tRNA(Pro)/Cys-tRNA(Cys) deacylase YbaK (EC 4.2.-.-) from *E. coli* | 159 |

**Table S2:** Location of ELR motifs in predicted aaRSs from fungal genomes.

| **Fungi** | **aaRS (predicted sub-cellular localization)** | **ORF** | **ELR location** |
| --- | --- | --- | --- |
| *A. niger* | GluRS (nucl) | 56891-mRNA | 73-75 |
| *A. niger* | LeuRS (cyto) | 52554-mRNA | 68-70 |
| *A. niger* | HisRS (mito) | 51854-mRNA | 150-152 |
| *C. albicans* | HisRS (mito) | orf19.4051 | 64-66 |
| *C. albicans* | LysRS (nucl) | orf19.6749 | 80-82 |
| *C. albicans* | GluRS (mito) | orf19.2415 | 152-154 |
| *C. neoformans* | TyrRS (mito) | CNBJ0260 | 117-119 |
| *C. neoformans* | ProRS (mito) | CNBB0150 | 113-115 |

**Figure S1:** Predicted signal sequence in fungal aaRSs based on MitoProt and TargetP servers.

**Predicted signal sequence in mitochondrial aaRSs from *A. niger***

GluRS 121468-mRNA SHRLRPATFIWDLCGRLCSITFWRSARE 28

IleRS 212883-mRNA MSSSRPSPQAQIYPGKGLGFISMLTLGASLHNVLSRV 37

LeuRS 183116-mRNA MQSLLRFRLSPTRSALPHPARWNCASRPILRPTAVPSRY 39

TrpRS 209919-mRNA MHIKISRYSLILTKAHRPRSIQPQLEVVSRQPNSSSFRR 39

TyrRS 54566-mRNA MRPHTGALPRALRLHVPKTLPQKTLCGARNYVRTAEVL 38

AsnRS 210632-mRNA MLTWRRTFATSVARLNNRPAARS 23

AlaRS 51231-mRNA MLQPTSRLMRIHRFIKEGELALTHLSSPFRSQPRTF 36

AspRS 57039-mRNA MYLARAVRCPPHLRATYVEISNYFRGRQLGRLSALQ 36

GlyRS 57294-mRNA MSHCSSAQRLGGLRCRRALGPSFRSFCPRHPNRLLHRQLLAFRP

PPTCASSSSFLPPRSFSTSLPRT 67

HisRS 51854-mRNA MHPSLQPRILRSARRALTECPWAPASSCRRS 31

PheRS 55517-mRNA MRLFATGRALRASSARW 17

**Predicted signal sequence in mitochondrial aaRSs from *C. albicans***

CysRS orf19.4931 MNLRFFLPKRIIRMSSTTT 19

GluRS orf19.2415 MKLSLPIIRNY 11

IleRS orf19.2382 MLRFKSKVRTFATSLVGL 18

TyrRS orf19.109 MLKTHTRNIPLIRRLARFNSTIARD 25

ValRS orf19.1295 MLYSRNLLLSTSSILFRKRIDFCIRRKLLSSSSII 35

AsnRS orf19.6698 LIQSRFPLKALQQVSSKRFNSSSLNPTIKNQLSNPPPIGSIIEARGF

VKSIRSS 55

AspRS orf19.4478 MLSFNVVSRDHTIVMLKILSRIQQHGVRYY 30

PheRS orf19.2039 MLKLPYRPVFCTLKQFRLY 19

HisRS orf19.4051 *Could not be predicted*

**Predicted signal sequence in mitochondrial aaRSs from *C. neoformans***

GluRS CNBK2470 MTRPLRPLLKVLSPRLGIRHYHSHHSSPQTTNVSSEIPKSARLRF 45

IleRS CNBN1610 MPSFIRPPTLLPRLNACRRFATPVSSSTSILQARF 35

TrpRS CNBJ3070 MPPRHRLLKALSFSFSLTRRAY 22

TyrRS CNBJ0260 MVLIRLSVSKRGAHLFHRK 19

ValRS CNBB3840 MLRHALRSVQRILTPSPIVYPRLQRTY 27

AlaRS CNBF2180 MLFGSVSTRSITSHFKFAPRLLSKTSFTV 29

AsnRS CNBD2410 MRSTQRRLSSLPPTIRSLLSSRRST 25

AspRS CNBK1200 MAFRASRTLISLRVSSRSTVARIAPRVWGAAASRVHIRG 39

HisRS CNBC0810 MLRPPIFRTLRRA 13

LysRS CNBH3030 MLRAVIRTATSTRQATTYAPKFKPQIPLRRTFIMSAPAA 39

PheRS CNBC0600 MAALVSLLRARPGPRLPVAARATLATRL 28

ProRS CNBB0150 MLRAFFKHSRAFITTRHHI 19

ThrRS CNBB5220 MLRRLIPHAARFSHRTPTRYTYPLLQAART 30

**Figure S2:** **(A)** Schematic representation of genomics and transcriptomic data for the VRS-Brix fusion protein in *A. niger*.

(http://www.broadinstitute.org/annotation/genome/aspergillus_group/MultiHome.html)

**(B)** Superimposition of crystal structure of probable ribosomal biogenesis protein from *Aeropyrum pernix* (Blue, PDB ID 2CXH) and modeled structure for Brix domain from *C. neoformans* YRS (Magenta, ID 194077-mRNA). The two proteins share ~16% sequence identity - however their folds are very similar as evident from the high confidence score (~99%) using the Phyre2 server.

**A**


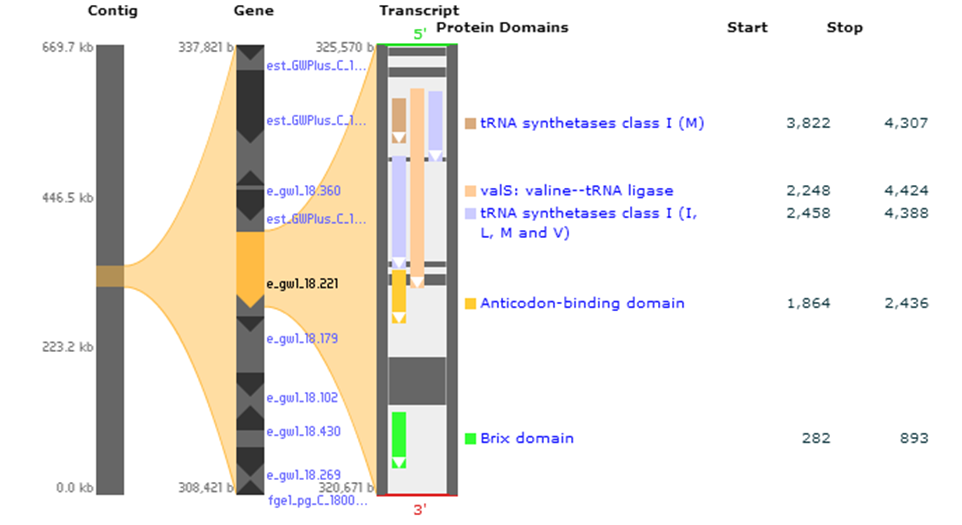


**B**


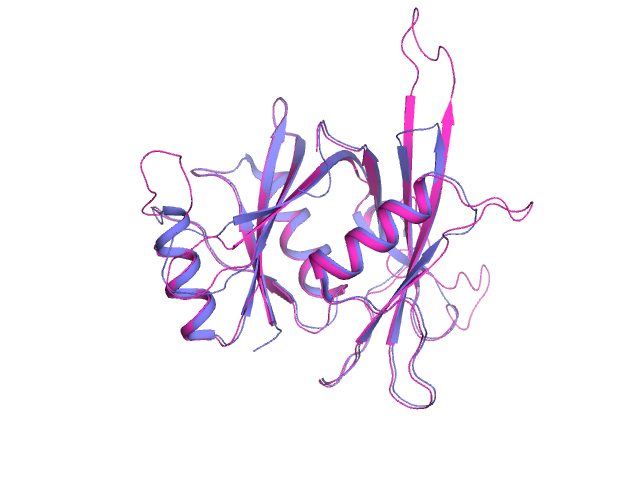


**Figure S3:** Schematic representation of genomics and transcriptomic data for the YRS-SAICAR fusion protein in *C. neoformans*.

(http://www.broadinstitute.org/annotation/genome/cryptococcus_neoformans/MultiHome.html)


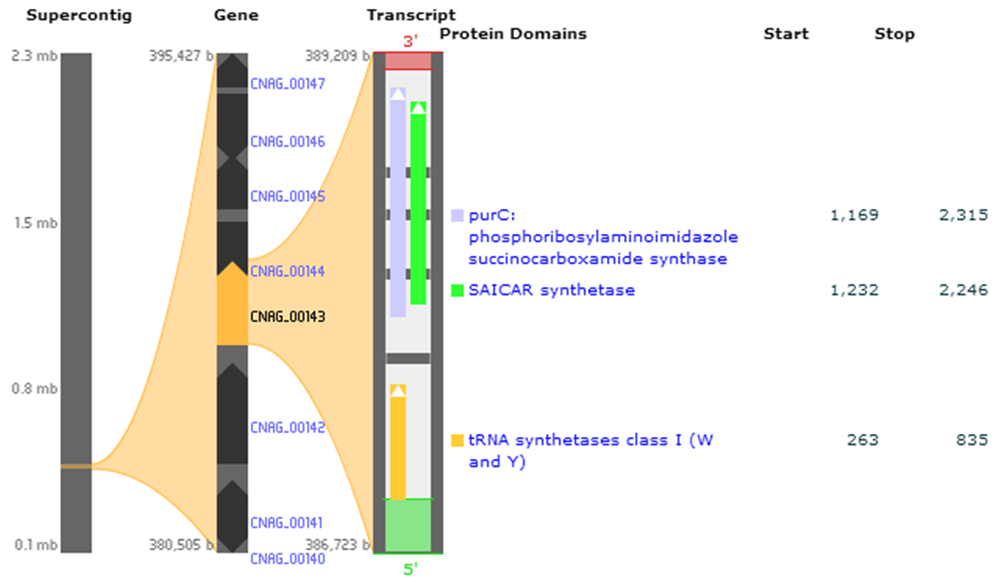

Supplement: Supplementary file 1 — Additional file 1: Table S1 and Table S2: Having the details of the dataset used for analyses and summary of fungal aaRSs with ELR motif, respectively. Figure S1. Shows details for signal sequences identified in fungal aaRSs. Figure S2. Shows (A) schematic representation for genome and transcriptome sequencing analyses for VRS-Brix and (B) modelling for Brix domain from C. neoformans. Figure S3. Schematic representation for genome and transcriptome sequencing analyses for YRS-SAICAR fusion protein in C. neoformans. (DOCX 356 KB) [file 12864_2014_6770_MOESM1_ESM.docx]
